# Prevalence and mechanisms of ciprofloxacin resistance in *Escherichia coli* isolated from hospitalized patients, healthy carriers, and wastewaters in Iran

**DOI:** 10.1186/s12866-023-02940-8

**Published:** 2023-07-17

**Authors:** Zohreh Neyestani, Farzad Khademi, Roghayeh Teimourpour, Mojtaba Amani, Mohsen Arzanlou

**Affiliations:** 1 Department of Microbiology, School of Medicine, Ardabil University of Medical Sciences, Ardabil, Iran; 2 Department of Medicinal Chemistry, Faculty of Pharmacy, Ardabil University of Medical Sciences, Ardabil, Iran

**Keywords:** *Escherichia coli*, Ciprofloxacin, QRDR, Antibiotic resistance, PMQR, Patients, Healthy carriers, Wastewater

## Abstract

**Background:**

This study was aimed to evaluate the prevalence and molecular characteristics of ciprofloxacin resistance among 346 *Escherichia coli* isolates collected from clinical specimens (*n* = 82), healthy children (*n* = 176), municipal wastewater (*n* = 34), hospital wastewater (*n* = 33), poultry slaughterhouse wastewater (*n* = 12) and livestock (*n* = 9) slaughterhouse wastewater in Iran.

**Methods:**

Ciprofloxacin minimum inhibitory concentration (MIC) was determined by agar dilution assay. Phylogroups and plasmid-mediated quinolone resistance (PMQR) genes were identified using PCR. Mutations in *gyrA*, *gyrB*, *parC,* and *parE* genes and amino acid alterations were screened through sequencing assay. The effect of efflux pump inhibitor (PAβN) on ciprofloxacin MICs in ciprofloxacin-resistant isolates was investigated using the microdilution method.

**Results:**

In total, 28.03% of *E. coli* isolates were phenotypically resistant to ciprofloxacin. Based on sources of isolation, 64.63%, 51.51%, 33.33%, 14.70%, 10.22% and 8.33% of isolates from clinical specimens, hospital wastewater, livestock wastewater, municipal wastewater, healthy children and poultry wastewater were ciprofloxacin-resistant, respectively. Eighty-one point eighty-one percent (Ser-83** → **Leu + Asp-87 → Asn; 78.78% and Ser-83 → Leu only; 3.03% **(**of ciprofloxacin-resistant *E. coli* isolates showed missense mutation in GyrA subunit of DNA gyrase, while no amino-acid substitution was noted in the GyrB subunit. DNA sequence analyses of the ParC and ParE subunits of topoisomerase IV exhibited amino-acid changes in 30.30% (Ser-80 → Ile + Glu-84 → Val; 18.18%, Ser-80 → Ile only; 9.10% and Glu-84 → Val only; 3.03%0 (and 15.38% (Ser-458 → Ala) of ciprofloxacin-resistant *E. coli* isolates, respectively. The PMQR genes, *aac(6')-Ib-cr*, *qnrS*, *qnrB*, *oqxA*, *oqxB,* and *qepA* were detected in 43.29%, 74.22%, 9.27%, 14.43%, 30.92% and 1.03% of ciprofloxacin-resistant isolates, respectively. No isolate was found to be positive for *qnrA* and *qnrD* genes. In isolates harboring the OqxA/B efflux pump, the MIC of ciprofloxacin was reduced twofold in the presence of PAβN, as an efflux pump inhibitor. The phylogroups B_2_ (48.45%) and A (20.65%) were the most predominant groups identified in ciprofloxacin-resistant isolates.

**Conclusions:**

This study proved the high incidence of ciprofloxacin-resistant *E. coli* isolates in both clinical and non-clinical settings in Iran. Chromosomal gene mutations and PMQR genes were identified in ciprofloxacin resistance among *E. coli* population.

## Background

*Escherichia coli* is the most common gram-negative rod responsible for a variety of intestinal and extraintestinal infections worldwide [1]. As a major part of the natural human intestinal microbial flora, *E. coli* is associated with a variety of community- and hospital-acquired opportunistic infections such as urinary tract infections (UTIs), septicemia, pneumonia, peritonitis, neonatal meningitis, and some other diseases [1]. Commonly, beta-lactam antibiotics are used to treat infections caused by *E. coli*. However, currently, due to increased resistance to β-lactam antibiotics, fluoroquinolones are used as alternative drugs to treat urinary tract infections caused by *E. coli* particularly in Asian countries [2]. These synthetic antibiotics inhibit the activity of DNA gyrase and topoisomerase IV, and break down the DNA strands, thereby killing the bacteria [3]. Resistance to fluoroquinolones in gram-negative bacteria is acquired either vertically by mutations in chromosomal genes or by horizontal transfer of resistance plasmids [4].

Mutations in genes encoding the target enzymes reduce the affinity of fluoroquinolones to bind to the DNA-enzyme complex and confer high levels of resistance to fluoroquinolones [5]. Mutations occur in the GyrA and GyrB subunits of DNA gyrase and the ParC and ParE subunits of topoisomerase IV enzymes [3]. Mutations in genes encoding outer membrane porins, e.g., OmpF, and efflux pumps, e.g., AcrAB-TolC, have also been shown in *E. coli*. These mutations reduce the expression of porins and increase the expression of efflux pumps, which decreases the intracellular concentration of antibiotics [6].

Plasmid-mediated quinolone resistance (PMQR) mechanisms include (i) Qnr proteins family including QnrA, QnrB, QnrD, QnrC, and QnrS, which protect gyrase and topoisomerase IV from fluoroquinolone inhibition. (ii) A new aminoglycoside acetyltransferase enzyme, AAC(6')-Ib-cr, which in addition to resistance to aminoglycosides, can acetylate fluoroquinolones [4]. (iii) Plasmid-dependent efflux pumps such as OqxA/B and QepA which extrude antibiotics out of the cell [7].

As mentioned above, the accumulation of mutations in bacterial DNA gyrase and topoisomerase IV is the main mechanism of resistance to fluoroquinolones in gram-negative bacteria [8]. Plasmid-mediated mechanisms usually confer low-level fluoroquinolone resistance which can lead to the occurrence of selective pressure for the growth of higher-level resistant mutants in the presence of fluoroquinolones at therapeutic concentrations [9]. Moreover, plasmid-mediated resistance can be transferred horizontally among the *Enterobacteriaceae* family, which could further facilitate the dissemination of antibiotic-resistance genes within different reservoirs [10].

Fluoroquinolones are widely used as antibiotics in human and veterinary medicine, and also as growth promoters in food-producing animals which lead to an increased prevalence of fluoroquinolone-resistant bacteria [11]. In *Enterobacteriaceae* family members particularly in *E. coli* isolates, the fluoroquinolone resistance is becoming increasingly common both in hospital- and community-acquired infections [2, 12]. However, the dissemination of fluoroquinolone-resistant *E. coli* isolates is not limited to clinical infections but also has been reported in various non-clinical resources [13].

Understanding the prevalence of ciprofloxacin resistance and elucidating the resistance genetic mechanisms would enable better decisions in treating *E. coli* infections and applying effective infection control measures.

Because of the lack of information on ciprofloxacin resistance in *E. coli* isolates especially in isolates derived from non-clinical settings in Iran, this study aimed: (i) to investigate the frequency of ciprofloxacin resistance in *E. coli* strains isolated from both clinical and non-clinical settings (healthy carriers, municipal, hospital, poultry and livestock wastewaters). (ii) to explore the genetic background behind ciprofloxacin resistance and (iii) to elucidate the molecular epidemiology of ciprofloxacin-resistant isolates using a phylogenetic grouping approach.

## Results

In the current study, 28.03% (*n* = 97) of *E. coli* isolates showed MICs above the resistance breakpoint (≥ 1 μg/mL) and were considered ciprofloxacin-resistant (Table 1). Clinical *E. coli* isolates with 64.63% (*n* = 53/82) showed the highest frequency of ciprofloxacin resistance followed by isolates from hospital wastewater (51.51%, *n* = 17/33), livestock slaughterhouse wastewater (33.33%, *n* = 3/9), municipal wastewater (14.7%, *n* = 5/34), healthy carriers (10.22%, *n* = 18/176) and poultry slaughterhouse wastewater (8.33%, *n* = 1/12). However, in comparison, there were no significant differences in the rate of ciprofloxacin resistance in isolates collected from clinical specimens with hospital wastewater (*P* > 0.05), healthy carriers with municipal wastewater (*P* > 0.05), and poultry slaughterhouse wastewater with livestock slaughterhouse wastewater (*P* > 0.05).

**Table 1:** MICs of ciprofloxacin for *E. coli* strains according to the isolation source

MIC (µg/mL)	Clinical specimens *N* = 82, n (%)	Healthy carriers *N* = 176 n (%)	Hospital wastewater *N* = 33 n (%)	Municipal wastewater *N* = 34, n (%)	Livestock slaughterhouse wastewater *N* = 9, n (%)	Poultry slaughterhouse wastewater *N* = 12, n (%)	Total *N* = 346, n (%)
0.25	29 (35.3)	(89.8) 158	(48.5) 16	(85.2) 29	6 (6.66)	(91.7) 11	(71.9) 249
0.5	0 (0.0)	0 (0.0)	0 (0.0)	0 (0.0)	0 (0.0)	0 (0.0)	0 (0.0)
1^a^	0 (0.0)	0 (0.0)	0 (0.0)	0 (0.0)	0 (0.0)	0 (0.0)	0 (0.0)
2	0 (0.0)	(2.9) 5	0 (0.0)	0 (0.0)	0 (0.0)	0 (0.0)	(1.5) 5
4	4 (4.9)	(4.6) 8	(3.0) 1	(60) 3	0 (0.0)	0 (0.0)	(4.6) 16
8	(2.4) 2	(0.5) 1	0 (0.0)	(40) 2	0 (0.0)	0 (0.0)	(1.5) 5
16	(6.0) 5	(0.5) 1	(18.2) 6	0 (0.0)	0 (0.0)	0 (0.0)	(3.5) 12
32	(19.6) 16	(0.5) 1	(15.2) 5	0 (0.0)	(33.3) 3	(8.3) 1	(7.5) 26
64	22 (26.9)	0 (0.0)	(12.1) 4	0 (0.0)	0 (0.0)	0 (0.0)	26 (7.5)
128	(1.2) 1	(1.1) 2	(3.0) 1	0 (0.0)	0 (0.0)	0 (0.0)	(1.2) 4
256	(3.7) 3	0 (0.0)	0 (0.0)	0 (0.0)	0 (0.0)	0 (0.0)	(0.8) 3
MIC_50_ (µg/mL)	32	0.25	0.25	0.25	0.25	0.25	0.25
MIC_90_ (µg/mL)	64	0.25	64	4	32	0.25	64

^a^The resistance breakpoint of ciprofloxacin against *E. coli*

Overall, the MICs of ciprofloxacin for ciprofloxacin-resistant *E. coli* isolates were between 2 and 256 μg/mL (Table 1). The isolates collected from clinical specimens, hospital wastewater, poultry slaughterhouse wastewater, and livestock slaughterhouse wastewater showed an MIC_50_ value of 32 µg/mL for each one, while for isolates collected from healthy carriers and municipal wastewater, the MIC_50_ value was 4 µg/mL.

**Fig. 1:**
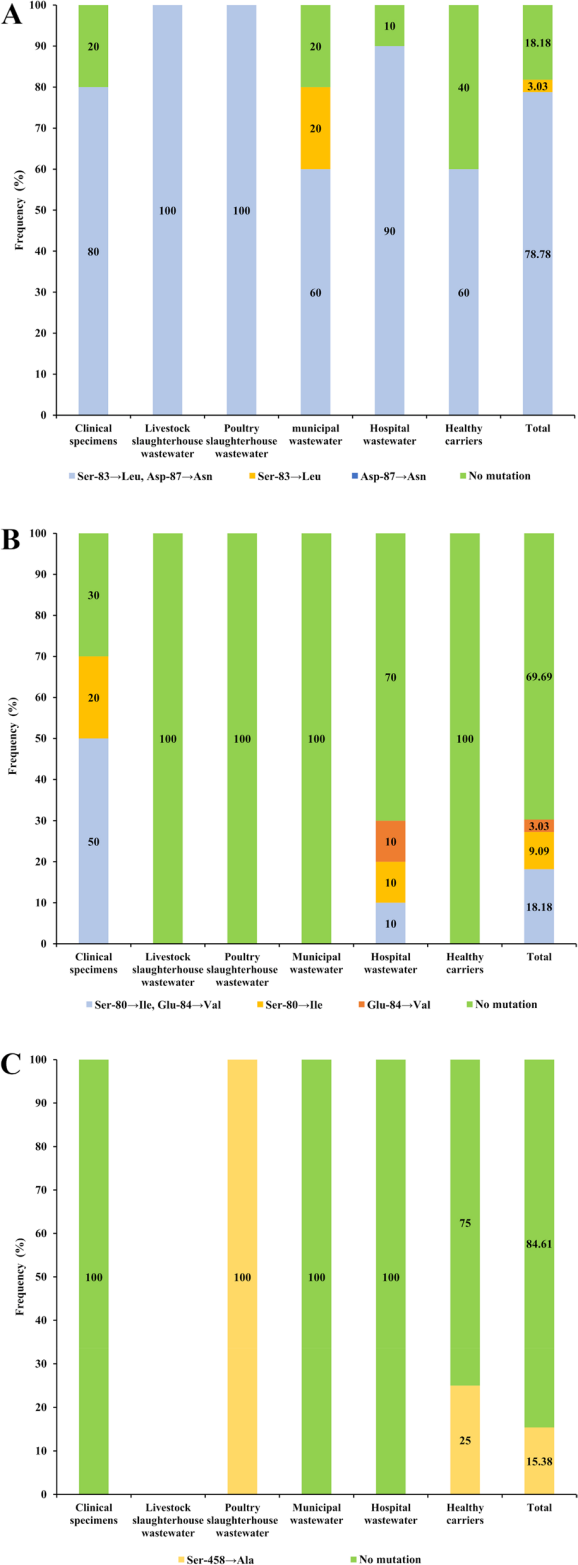
Frequency distribution of missense mutations in (**A**) *gyrA*, (**B**) *parC*, and (**C**) *parE* genes in ciprofloxacin-resistant *E. coli* isolates according to the isolation source

In the current study, as shown in Figure 1A, 81.81% (*n* = 27/33) of ciprofloxacin*-*resistant *E. coli* isolates showed at least a missense mutation in the *gyrA* gene. Among them, 78.78% (*n* = 26/33) of isolates had double amino-acid substitution at sites 83 (Ser-83→Leu) and 87 (Asp-87→Asn), and 3.03% (*n* = 1/33) of isolates showed a single substitution at site 83 (Ser-83→Leu) in GyrA subunit of DNA gyrase enzyme. Statistically, there was no association between the types of mutation in the *gyrA* gene and the sources of the isolates (*P* > 0.05). In this study, no missense mutation was identified in *gyrB.*

DNA sequence analyses of the ParC subunit of topoisomerase IV exhibited amino-acid changes in 30.30% (*n* = 10/33) of ciprofloxacin-resistant *E. coli* isolates. Among them, 18.18% (*n* = 6/33) of isolates showed double amino-acid substitution at positions 80 (Ser-80→Ile) and 84 (Glu-84→Val). Additionally, 9.09% (*n* = 3/33) and 3.03% (*n* = 1/33) of ciprofloxacin-resistant *E. coli* isolates harbored single substitutions at positions 80 (Ser-80→ Ile) and 84 (Glu -84→Val), respectively (Fig. 1B). These changes were significantly associated with isolates from clinical specimens and hospital wastewater.

In the present study, we identified 15.38% of isolates with a single missense mutation in ParE subunit of topoisomerase IV, which encodes Ser-458→Ala (Fig. 1C). Statistically, there was no association between the types of mutation in *parE* gene and sources of the isolates (*P* > 0.05).

The isolates with single or no mutation in topoisomerase IV and DNA gyrase showed lower ciprofloxacin MIC_50_ and MIC_90_ values (Table 2).

**Table 2:** Association between missense mutations in GyrA, ParC, and ParE subunits and ciprofloxacin MIC levels in ciprofloxacin-resistant *E. coli* isolates

	Amino acid substitution			Isolates *N* = 33 n (%)	MIC_50_ µg/mL	MIC_90_ µg/mL
Profile	GyrA	ParC	ParE^a^			
1	Ser-83 → Leu, Asp-87 → Asn	Ser-80 → Ile	ND	3	64	128
2	Ser-83 → Leu, Asp-87 → Asn	Ser-80 → Ile, Glu-84 → Val	-^b^	2	8	32
3	Ser-83 → Leu, Asp-87 → Asn	Ser-80 → Ile, Glu-84 → Val	ND	4	64	128
4	Ser-83 → Leu, Asp-87 → Asn	Glu-84 → Val	ND	1	64	64
5	Ser-83 → Leu, Asp-87 → Asn	-	ND	10	32	64
6	Ser-83 → Leu, Asp-87 → Asn	-	-	4	4	8
7	Ser-83 → Leu, Asp-87 → Asn	-	Ser-458 → Ala	2	32	128
8	Ser-83 → Leu	-	-	1	4	4
9	-	-	ND	2	16	32
10	-	-	-	4	4	32

^a^For the ParE subunit, 13 isolates were examined

^b^Identical to wild type

ND: not determined

The prevalence of PMQR genes is described in Table 3. Overall, the *aac (6')-Ib-cr* gene was detected in 43.29% (*n* = 42/97) of ciprofloxacin-resistant isolates. *qnrS* and *qnrB* genes were identified in 74.22% (*n* = 72/97) and 9.27% (*n* = 9/97) of ciprofloxacin-resistant *E. coli* isolates, respectively. In this study, no isolate was found to be positive for *qnrA* and *qnrD* genes. The genes encoding efflux pumps; *oqxB, oqxA,* and *qepA* were identified in 30.92% (*n* = 30/97), 14.43% (*n* = 14/97), and 1.03% (*n* = 1/97) of ciprofloxacin-resistant *E. coli* isolates, respectively. The MIC_50_ values for ciprofloxacin in isolates harboring *aac (6')-Ib-cr* and *qepA* were higher than those containing other genes. Overall, MIC_90_ values were high in all isolates except for those containing *qnrB* (Table 3)*.*

**Table 3:** Occurrence of PMQR genes and ciprofloxacin MIC levels in ciprofloxacin*-*resistant *E. coli* isolates collected from different sources

Source	PMQR encoding genes							
	*aac (6')-Ib-cr*	*qnrS*	*qnrB*	*qnrD*	*qnrA*	*oqxB*	*oqxA*	*oqxA*
Livestock slaughterhouse wastewater *N* = 3, n (%)	2 (66.66)	1 (33.33)	0 (0.00)	0 (0.00)	0 (0.00)	1 (33.33)	0 (0.00)	0 (0.00)
Poultry slaughterhouse wastewater *N* = 1, n (%)	0 (0.00)	0 (0.00)	1(100)	0 (0.00)	0 (0.00)	0 (0.00)	0 (0.00)	0 (0.00)
Municipal wastewater *N* = 5, n (%)	0 (0.00)	5 (100)	3 (60)	0 (0.00)	0 (0.00)	0 (0.00)	0 (0.00)	0 (0.00)
Hospital wastewater *N* = 17 n (%)	7 (41.17)	9 (52.94)	1(5.88)	0 (0.00)	0 (0.00)	11 (64.70)	0 (0.00)	0 (0.00)
Healthy carriers *N* = 18, n (%)	1 (5.55)	9 (50)	0 (0.00)	0 (0.00)	0 (0.00)	12 (66.66)	9 (50)	1 (5.55)
Clinical specimens *N* = 53, n (%)	32 (60.37)	48 (90.56)	4 (7.54)	0 (0.00)	0 (0.00)	6 (11.32)	6 (11.32)	0 (0.00)
Total *N* = 97, n (%)	42 (43.29)	72 (74.22)	9 (9.27)	0 (0.00)		30 (30.92)	14 (14.43)	1 (1.03)
MIC_50_ (µg/mL)	64	32	16	-	-	16	4	128
MIC_90_ (µg/mL)	64	64	32	-	-	64	64	128

Analysis of ciprofloxacin resistance genes co-occurrences in ciprofloxacin-resistant *E. coli* isolates revealed 16 different patterns. Among them, 17.52% of isolates contained profiles with 3 different PMQR genes, simultaneously (Table 4). Increased levels of ciprofloxacin MICs were not observed in isolates containing multiple PMQR genes.

**Table 4:** Profiles of PMQR genes and ciprofloxacin MIC levels in ciprofloxacin-resistant *E. coli* isolates

Profile No	Gene combination	Gene No	Frequency *N* = 97, n (%)	Isolates *N* = 97, n (%)	MIC_50_ µg/mL	MIC_90_ µg/mL
1	*oqxB*	1	4 (4.12)	35 (36.08)	16	32
2	*aac (6’)-Ib-cr*		5 (5.15)		32	64
3	*qnrS*		25 (25.77)		32	64
4	*qepA*		1 (1.03)		128	128
5	*oqxB, qnrB*	2	1 (1.03)	41 (42.26)	16	16
6	*oqxA, oqxB*		5 (5.15)		64	128
7	*aac (6’)-Ib-cr, oqxA*		1 (1.03)		64	64
8	*aac (6’)-Ib-cr, oqxB*		3 (3.09)		32	64
9	*qnrS, qnrB*		4 (4.12)		4	32
10	*qnrS, oqxB*		9 (9.27)		4	32
11	*qnrS, aac (6’)-Ib-cr*		18 (18.55)		64	128
12	*aac (6’)-Ib-cr, oqxA, oqxB*	3	1 (1.03)	17 (17.52)	2	2
13	*qnrS, oqxB, oqxA*		2 (2.06)		2	4
14	*aac (6’)-Ib-cr, oqxA, qnrS*		5 (5.15)		64	256
15	*qnrS, aac (6’)-Ib-cr, qnrB*		4 (4.12)		16	32
16	*qnrS, aac (6’)-Ib-cr, oqxB*		5 (5.15)		32	64

As shown in Table 5, in isolates harboring OqxA/B efflux pumps the MIC of ciprofloxacin was reduced twofold in the presence of PAβN (the efflux pump inhibitor) compared to the absence of inhibitor. No change in MIC of ciprofloxacin was observed in isolates lacking *oqxA/B* genes in the presence of PAβN.

**Table 5:** Effect of PaβN (phenylalanine-arginine beta-naphthylamide) on MICs of ciprofloxacin in selected ciprofloxacin-resistant *E. coli* isolates

	Molecular characteristics of the isolates				MIC (µg/mL) without PAβN	MIC (µg/mL) with PAβN
	Gene pattern	Mutations				
		*par C*	*gyr B*	*gyr A*		
1	*oqxA*/*B*	**-**	**-**	** + **	32	16
2	*oqxA*/*B*	**-**	**-**	**-**	128	64
3	*oqxA/B*	**-**	**-**	**-**	128	64
4	*oqxA*/*B*	**-**	**-**	**-**	64	32
5	*oqxA*/*B*	**-**	**-**	**-**	16	8
6	no *oqxA*/*B*	-	-	+	8	8
7	no *oqxA*/*B*	-	-	-	256	256

**Fig. 2:**
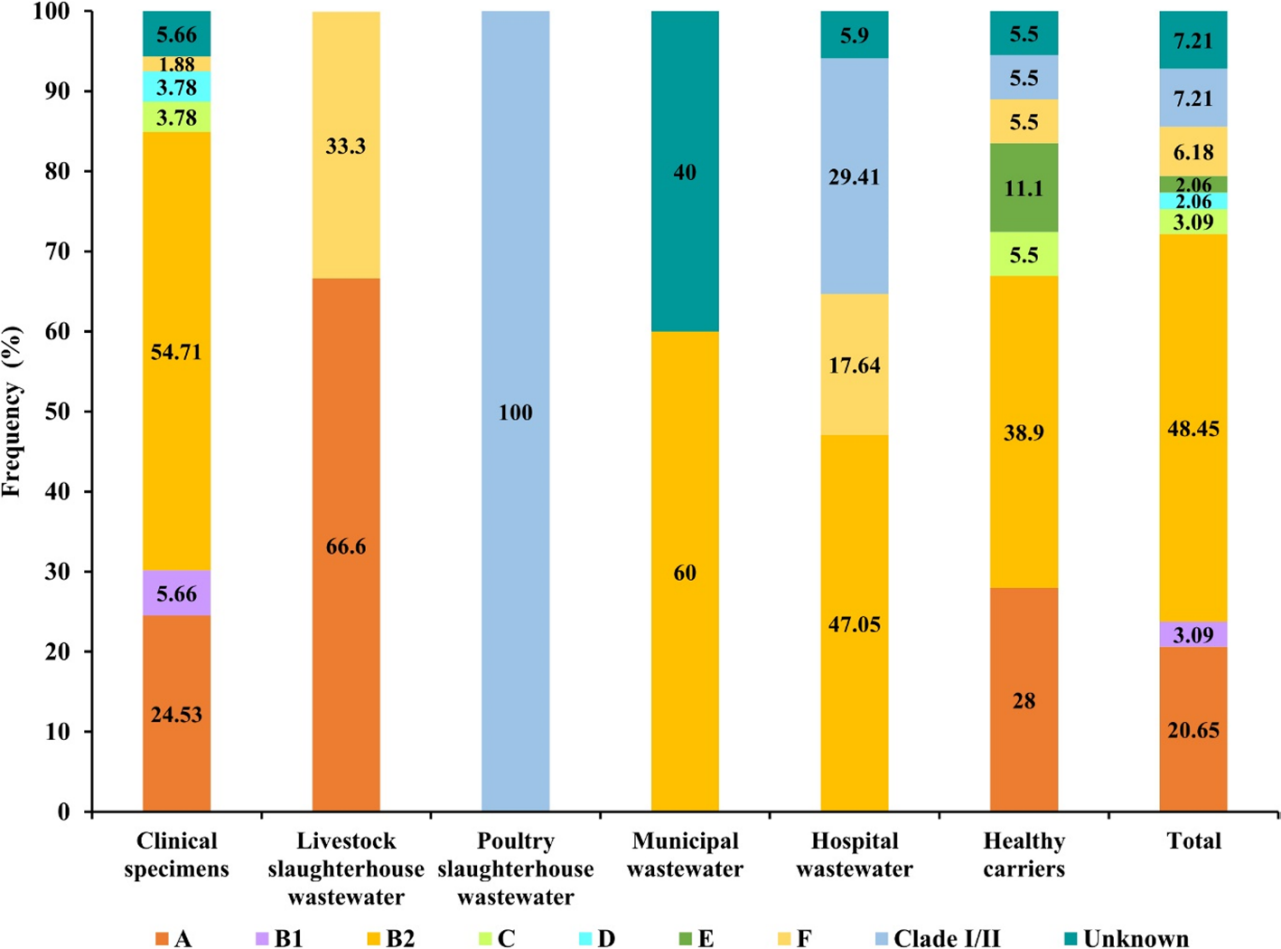
Distribution of the phylogroups among ciprofloxacin-resistant *E. coli* isolates according to the isolation source

In the present study, as shown in Figure 2, ciprofloxacin-resistant *E. coli* isolates were distributed among different phylogroups. However, irrespective of the source of collection, phylogroups B_2_ and A with 48.45% (*n* = 47/97) and 20.65% (*n* = 20/97) of the isolates were the most predominant groups identified, respectively. According to the source of collection, the incidence of phylogroup B_2_ was significantly higher than other groups among strains isolated from clinical specimens; 54.71% (*n* = 29/53), municipal wastewater; 60% (*n* = 3/5), hospital wastewater; 47.05% (*n*= 8/17) and healthy people; 38.90% (*n* = 7/18). In isolates collected from livestock wastewater, the occurrence of strains belonging to phylogroup A was significantly higher and a single ciprofloxacin-resistant *E. coli* isolate collected from poultry wastewater belonged to clade I/II phylogroup.

The relative frequency distribution of PMQR genes among ciprofloxacin-resistant *E. coli* phylogroups was significantly different (Table 6). *qnrS* and *oqxA* genes were most frequently detected in phylogroup D, *oqxB* in phylogroup E, *qepA* in phylogroup B_2,_ and *qnrB* in isolates with unknown phylogroup.

**Table 6:** Association between frequency of PMQR genes and phylogroups in ciprofloxacin-resistant *E. coli* isolates

Genes										
	A *N* = 20	B_1_ *N* = 3	B_2_ *N* = 47	C *N* = 3	D *N* = 2	E *N* = 2	F *N* = 6	Clade I/II *N* = 7	Unknown *N* = 7	*P* value
*qnrS*, n (%)	15 (75)	2 (66.6)	38 (80.8)	1 (33.3)	2 (100)	0 (0.0)	4 (66.6)	5 (71.4)	5 (71.4)	0.001
*qnrB*, n (%)	1 (5)	0 (0.0)	3 (6.3)	0 (0.0)	0 (0.0)	0 (0.0)	1 (16.6)	1 (14.2)	3 (42.8)	0.001
*oqxB,* n (%)	5 (25)	0 (0.0)	15 (31.9)	1 (33.3)	0 (0.0)	2 (100)	3 (50)	2 (28.5)	2 (28.5)	0.001
*oqxA*, n (%)	4 (20)	0 (0.0)	7 (14.8)	1 (33.3)	1 (50)	0 (0.0)	0 (0.0)	0 (0.0)	1 (14.2)	0.001
*qepA, *n (%)	0 (0.0)	0 (0.0)	1 (2.12)	0 (0.0)	0 (0.0)	0 (0.0)	0 (0.0)	0 (0.0)	0 (0.0)	0.001

## Discussion

Ciprofloxacin, a fluoroquinolone antibiotic, has been widely used to treat infections caused by *E. coli* [14]. Hence, *E. coli* resistance to ciprofloxacin has been steadily increasing worldwide [14]. Previous reports in Iran indicated ciprofloxacin resistance varying from 30 to 100% in *E. coli* isolates collected from clinical specimens [15, 16]. Our results in this study also confirmed a high prevalence of ciprofloxacin-resistant *E. coli* clinical isolates in Ardabil hospitals (64.6%). The high resistance rate of *E. coli* clinical isolates can be attributed to the high consumption of ciprofloxacin in Iranian hospitals [17, 18]. Additionally, geographical differences in the prevalence of resistance to ciprofloxacin can be due to the extent of use of fluoroquinolone in each region or differences in methods of the assessment of antibiotic resistance [15].

Notably, the prevalence of resistance to ciprofloxacin in *E. coli* isolates in healthy children (10.22%) was lower than in clinical isolates. A study reported a higher incidence of colonization with ciprofloxacin-resistant *E. coli* in Spanish healthy adults (24%) and children (16%) [19, 20]. Exposure of commensal flora to antibiotics is a known risk factor correlated with increased antimicrobial resistance rate [21]. The high prevalence of multidrug-resistant commensal *E. coli* isolated from healthy individuals is being reported from different regions, especially in low- and middle-income countries [22]. The widespread exposure of commensal *E. coli* to ciprofloxacin is not the case in the current study because fluoroquinolones are not frequently prescribed in outpatient settings in Iran [23]. Similarly, resistance to ciprofloxacin in commensal *E. coli* isolates was reported in children with no previous exposure to ciprofloxacin [12]. Healthy children may acquire ciprofloxacin*-*resistant *E. coli* from adults or through foods and environmental contaminations.

In wastewater resources, the rate of resistance to ciprofloxacin in *E. coli* strains isolated from hospital wastewater was significantly higher than in isolates collected from other resources (51.5%). Similar results were reported from the studies in Hamadan (30.61%), Tabriz (29%), and the Netherlands (54%) [24–26]. In the current study, 14.70% of *E. coli* isolates from municipal wastewater were found to be ciprofloxacin-resistant. These findings are somewhat akin to the profile obtained in fecal isolates from healthy individuals, discussed above. The bacterial profile of untreated municipal wastewater has been shown to mirror that of human fecal flora [27], in a way that municipal wastewater isolates can be used as a surrogate to study human commensal *E. coli* in a local population [28, 29]. In a study in Iran, it has been shown that fluoroquinolones accounted for 5.7% of the antibiotics sold out by veterinary pharmaceutical companies in 2010 [30], which can promote the emergence of resistance to ciprofloxacin in bacteria from food-producing animals [31]. However, in countries where fluoroquinolones are not permitted for use in food-producing animals, ciprofloxacin resistance has also not been observed in bacteria of animals [32]. In the present study, 33.3% of isolates from livestock slaughterhouse wastewater and 8.3% of poultry slaughterhouse wastewater isolates were resistant to ciprofloxacin, which is in accordance with the results of the study by Naraghi et al. in northeast Iran [33].

Point mutations in the chromosomal target genes (*i.e, gyrA*, *gyrB*, *parC,* and *parE* genes) are the most common bacterial resistance mechanisms to quinolones, which missense amino-acid substitutions occur at several sites in the QRDR region of target proteins [8]. It has been documented that the accumulation of specific mutations in both the DNA gyrase and topoisomerase genes causes high resistance to ciprofloxacin in *E. coli* isolates [8], accordingly similar results were observed in the current study. However, a single mutation in one of these genes is often sufficient to increase the ciprofloxacin MIC beyond the resistance breakpoints, hence allowing the emergence of secondary mutations in the presence of ciprofloxacin selective pressure, that further increase the MIC level [8]. Most reported point mutation in the *gyrA* gene occurs in nucleotides 248 and 260 changing serine-83 and asparagine-87 amino acids [34]. Similarly, Ser-83→Leu and Asp-87→Asn amino-acid substitutions in the GyrA subunit were the most identified mutations in our isolates. Amino-acid changes in the GyrB subunit of DNA gyrase enzyme are relatively low [8, 35–39]. Likewise, in the present study, no missense mutation was observed in the GyrB subunit in *E. coli* strains resistant to ciprofloxacin.

The most common missense mutations in the *parC* gene are reported in nucleotides 238/239 and 250/251 leading to changes in serine-80 and glutamate-84 [34]. In the current study, the most common amino-acid changes in the ParC subunit were the substitution of glutamate to valine (E-84→V) (21.2%) and serine to isoleucine (S-80→I) (27.2%). Similar findings were reported from different regions of Iran [40–42] and other countries [36–39, 43]. In general, missense mutations in the QRDR region of ParE subunit in *E. coli* is infrequent compared to the ParC subunit of topoisomerase IV [8]. The sole replacement observed in the current study was Ser-458→Ala consistent with reports from other countries [42, 44–47].

Genes carried by a plasmid, such as *aac (6')-Ib-cr, qnr, qepA,* and *oqxA/B* contribute to ciprofloxacin resistance in *E. coli* as reported in bacteria from human, animal, and environmental resources [48]. The PMQR genes often confer low-level resistance to quinolones and/or fluoroquinolones by themselves, but instead, they create favorable conditions for the selection of more resistant mutants [9]. However, among different PMQR determinates some genes like *aac (6')-Ib-cr* and *qepA* are slightly associated with higher ciprofloxacin MIC values [10]. In accordance with previous reports, *E. coli* isolates containing these genes showed higher ciprofloxacin MIC_50_ values in our study. The *aac (6')-Ib-cr* is more commonly found in *E. coli* compared to other Enterobacteriaceae members [49]. We detected *aac (6')-Ib-cr* in 43.2% of ciprofloxacin-resistant *E. coli* isolates. In controversy to other studies that reported *qnrB* as the most frequent Qnr protein-encoding gene [50], we identified the *qnrS* gene in most ciprofloxacin-resistant *E. coli* isolates. Yanat *et. al* reported the distribution of the PMQR genes differs by geographic region and isolates selection criteria [48] which may explain the mentioned controversy. However, our results on the *qnr* genes pattern are inconsistent with the findings in Iran and some countries [8, 31, 37, 41, 51–53]. The *oqxA, oqxB,* and *qepA* genes encoding plasmid-mediated efflux pumps were identified in 14.43%, 30.92% and 1.03% of ciprofloxacin-resistant *E. coli* isolates, respectively. The QepA and OqxA/B are generally rare in Enterobacteriaceae members [48] meanwhile most OqxA/B cases were reported from animal isolates in China [54]. Surprisingly, *oqxA/B* positive ciprofloxacin-resistant *E. coli* isolates were from healthy children and hospital wastewater in this study. Similar to findings reported by Khalil, *et.al.* [55], we found that PaβN, as an efflux pump inhibitor, reduced the ciprofloxacin MIC in isolates containing OqxA/B supporting the importance of OqxA/B in ciprofloxacin resistance. It has been shown that the coexistence of PMQR determinates in *E. coli* could potentially cause higher levels of quinolone resistance [50]. However, such an association was not observed in our isolates. This controversy may be explained by the additional undetected resistance mechanisms such as overexpression of chromosomal ArcAB-TolC multi-drug efflux pump in ciprofloxacin-resistant *E. coli* isolates [6].

In addition to ciprofloxacin resistance data, we also provided relevant information on the molecular epidemiologic characteristics of ciprofloxacin-resistant *E. coli* isolates. *E. coli* is classified into eight phylogroups named A, B1, B_2_, C, D, E, F, and clade I/II [56]. Irrespective of collection sources, the phylogroup B_2_ (48.45%) and A (20.65%) were the predominant groups in ciprofloxacin-resistant *E. coli* isolates in the current study. It has been documented that the distribution of *E. coli* phylogroups differs according to geographic location, climate, specific lifestyles, and hosts [57]. We observed a positive correlation between the occurrences of phylogroups in isolates from clinical specimens with hospital wastewater, and healthy carriers with municipal wastewater. The phylogroup B_2_ was the most common group in isolates collected from the abovementioned sources. This may express the possibility of dissemination of ciprofloxacin-resistant *E. coli* isolates from hospital discharges and fecal materials from healthy children into hospital and municipal wastewaters, respectively [27]. This explanation is also supported by the fact that the ciprofloxacin resistance prevalence and MIC_50_ levels coincided in clinical and commensal isolates with those from hospital and municipal wastewaters, respectively.

Some shreds of evidence represent the higher antibiotic resistance rate in certain *E. coli* phylogroups compared to others [15]. Our literature surveys did not get any results on the association between phylogroups of *E. coli* with a specific antibiotic resistance phenotype. Here, we report that ciprofloxacin resistance in *E. coli* may link to phylogroup B_2_. The distribution of PMQR genes was significantly different among phylogroups. However, more diverse PMQR genes were observed in phylogroup B_2_ isolates. This is similar to the findings reported by Nojoomi et al., which showed a higher incidence of some beta-lactamase encoding genes and virulence determinants in phylogroup B_2_ isolates [58].

### Limitations of the study

Due to limited resources, the mutations in DNA gyrase and topoisomerase genes were not studied on all ciprofloxacin-resistant *E. coli* isolates. Additionally, the expression level of chromosomal multi-drug efflux pump ArcAB-TolC was not studied which could contribute to ciprofloxacin resistance.

We characterized the molecular relatedness of the ciprofloxacin-resistant *E. coli* isolates using the PCR-based phylotyping method. However, more robust molecular typing methods such as pulsed-field gel electrophoresis (PFGE) and multi-locus sequence typing (MLST) methods need to determine the isolates' precise genetic relatedness from different resources.

## Conclusion

To conclude, ciprofloxacin resistance was significantly prevalent in *E. coli* isolates from clinical specimens, healthy children, and wastewaters in Iran. Hence, continuous surveillance of ciprofloxacin resistance trends and MIC values along with prudent use of fluoroquinolone antibiotics in clinics, prohibition of its use in food-producing animals, and efficient disinfection of wastewater are recommended to prevent the emergence and spread of ciprofloxacin-resistant *E. coli* isolates. On the other hand, we confirmed the role of multiple mechanisms including the presence of PMQR genes and mutations in the QRDRs in the emergence of ciprofloxacin resistance in *E. coli* isolates from both clinical and non-clinical sources in Ardabil. Double and concurrent mutations within *gyrA* and *parC* genes were common and associated with increased ciprofloxacin MICs in ciprofloxacin-resistant isolates. Furthermore, *aac(6')-Ib-cr, qnrS,* and *oqxB* were the most prevalent PMQR genes in ciprofloxacin-resistant isolates, and the presence of *aac(6')-Ib-cr* and *qepA* genes were associated with higher ciprofloxacin MIC_50_ levels. Ciprofloxacin-resistant *E. coli* isolates were mainly linked to phylogroup B_2._ This suggests the possible dissemination of ciprofloxacin-resistant *E. coli* through various environments. Therefore, understanding antibiotic resistance mechanisms and the genetic relatedness of bacteria using reliable methods can help develop effective strategies to prevent the spread of resistant bacteria.

## Materials and methods

### Bacterial isolates

In this study, a total of 346 *E. coli* isolates from clinical specimens (*n* = 82), healthy children (*n* = 176) and municipal (*n* = 34), hospital (*n* = 33), poultry (*n* = 12) and livestock (*n* = 9) slaughterhouse wastewater were included. The isolates from healthy children [28] and wastewater resources were previously collected in Ardabil from April 2017 to February 2019. Wastewater samples had been collected from the raw sewage influent of four teaching hospitals (Imam, Fatemi, Alavi, and Bouali) affiliated with Ardabil University of Medical Sciences (ARUMS), and also poultry slaughterhouse, livestock slaughterhouse, and municipal wastewater treatment plants in Ardabil province, Iran. Liquid wastewater samples were collected from one sampling point in 500 mL sterile bottles in accordance with the U.S. Environmental Protection Agency (US EPA) standard operating procedure for wastewater sampling [59]. Collected samples were immediately transferred to the microbiology laboratory in cold box containers and kept at 4 °C. Microbiological analysis was performed in less than 2 h after sample collection. Clinical isolates were from patients admitted to a referral hospital (Imam) affiliated with ARUMS from April 2021 to September 2021. The study was approved by the regional ethics committee in ARUMS (reference no. IR.ARUMS.REC.1399.553). Isolates were identified using conventional microbiology and biochemical tests [60] and kept in 15 % glycerol stock cultures at – 70 °C for further studies.

### Ciprofloxacin susceptibility assay

The minimum inhibitory concentrations (MICs) of ciprofloxacin (Sigma-Aldrich, USA) for *E. coli* were determined using the agar dilution method [61]. The concentration of ciprofloxacin ranged from 0.12 to 256 µg/mL. The results were interpreted according to CLSI guidelines [62]. The isolates with MICs ≥1 µg/mL and 0.5 µg/mL were considered ciprofloxacin-resistant and -intermediate-resistant (I), respectively.

### Amplification of ciprofloxacin resistance encoding genes

Genomic DNA from ciprofloxacin-resistant *E. coli* isolates was extracted using the boiling method as previously reported [63]. The presence of PMQR encoding genes: *qnr* (*A, B, D,* and *S*), *aac (6')-Ib-cr*, *qep*A, and *oqx*A/B were detected by PCR using previously described primer sequences (synthesized in SinaClon Co. Iran) and cycling parameters [42, 56, 64, 65].

A representative PCR product for each gene was sequenced (Microsynth Co. Switzerland) and aligned using the Basic Local Alignment Search Tool (BLAST) at National Center for Biotechnology Information (NCBI) Center database [available at http://blast.ncbi.nlm.nih.gov/]. Genomic DNA from isolates carrying the corresponding resistance genes was used as a positive control in PCR experiments.

### Detection of missense mutations in topoisomerase enzymes

In total, 33 ciprofloxacin-resistant isolates were selected based on distribution frequency and MIC levels from hospital wastewater (*n* = 10), municipal wastewater (*n* = 5) livestock slaughterhouse wastewater (*n* = 2), poultry slaughterhouse wastewater (*n* = 1), healthy carriers (*n* = 5) and clinical isolates (*n* = 10). The *gyrA*, and *gyrB* genes encoding DNA gyrase and *parC* gene encoding topoisomerase IV enzymes were amplified using specific primers as described previously [42, 45]. For topoisomerase IV *parE* gene 13 isolates were included [45]. The amplified DNA fragments were sent for sequencing by Microsynth Co. Switzerland. Point mutations in *gyrA*, *gyrB*, *parC,* and *parE* genes were identified throughout the nucleotide sequences by comparing them with the corresponding sequence of *E. coli* ATCC 25922 using DNAMAN software version 10 [https://dnaman.software.informer.com/].

### Inhibition study of efflux pumps using phenylalanine -Arginine β- naphthylamide (PAβN)

To evaluate the role of OqxA/B efflux pumps in *E. coli* ciprofloxacin resistance, 5 isolates harboring *oqxA/B* genes were selected. The reduction in MICs of ciprofloxacin was evaluated using the microdilution method in 96-house plates in accordance with CLSI instructions [62]. Briefly, serially twofold dilutions of the ciprofloxacin, with concentrations ranging from 0.12 to 256 µg/mL, were prepared in a sterile Müller Hinton Broth (Himedia, India) culture medium. Then, 100 μL from each dilution was transferred into wells of a microtiter plate and inoculated with 5 µL of standardized bacterial suspension (1.5 × 10^7^ CFU/mL). Finally, a constant volume (4 μL) of the efflux pump inhibitor (PAβN) at a concentration of 100 μg/mL was added to each well. The plates were incubated at 37 °C for 24 h. The change of ciprofloxacin MIC in the presence of the inhibitor was recorded compared to those with no PAβN [66].

### Phylotyping of E. coli isolates

The phylogroups of ciprofloxacin-resistant *E. coli* strains were determined by Quadruplex-PCR as previously described by Clermont *et al.* In this method, based on the combined patterns of *arpA*, *chuA*, *yjaA*, TspE4.C2, *trpA*, *arpAgpE*, *trpAgpC,* and internal control genes, *E. coli* isolates are classified into groups A, B1, B_2_, C, D, E, F and clade I [56].

### Statistical analyses

Comparison of the prevalence of resistance to ciprofloxacin and association between resistance genes in *E. coli* isolates from clinical specimens, hospital wastewater, municipal wastewater, poultry slaughterhouse wastewater, and livestock slaughterhouse wastewater were evaluated using the Chi-square and Fisher’s exact tests. A *p*-value of < 0.05 was used to indicate statistically significant results.

## Data Availability

The datasets generated and analyzed during the current study are available in the NCBI GenBank repository, under the accession numbers: OM791372 to OM791375, MZ359353 to MZ359375, OM791362 to OM791371 and OM831129.

## Abbreviations

ATCC: American-type culture collection
BLAST: Basic local alignment search tool
CLSI: Clinical and laboratory standards institute
DNA: Deoxyribonucleic acid
EPA: Environmental protection agency
MIC: Minimum inhibitory concentration
NCBI: National Center for biotechnology information
PAβN: Phenylalanine -arginine β- naphthylamide
PCR: Polymerase chain reaction
PMQR: Plasmid-mediated quinolone resistance
